# Supplementary material for: Prevalence of emergency caesarean delivery and its predictors among women who give birth in Ethiopia using further analysis of EDHS 2016 data: A mixed effect model
Source: PLoS One. 2024 May 16;19(5):e0300528. doi: 10.1371/journal.pone.0300528 (PMC11098499; doi:10.1371/journal.pone.0300528)
Supplement: Attachment — Submitted filename: respose to reviewers.pdf [file pone.0300528.s002.pdf]

Prevalence of emergency cesarean delivery and its predictors among women who give birth in Ethiopia using further analysis of EDHS 2016 data: A mixed effect model

Muluken Chanie Agimas

1. College of Medicine and health science, institute of public health, Department of Epidemiology and Biostatistics, University of Gondar, Gondar, Ethiopia.

Authors address:

Muluken Chanie Agimas (MCA): mulukensrc12@gmail.com

To: PLOS ONE <em@editorialmanager.com>

Manuscript Number: PONE-D-23-35082

Cover Letter for reviewer's comments and authors responses

We would like to thank both the Editorial board members and reviewers for kind comments and suggestions. Accordingly, we have tried to correct and respond to those comments point by point, page by page, and line by line. Therefore, we prepared this author's response, the revised manuscript, the separate revised figures and tables, and the tracked changes of the manuscript.

Sincerely, with best regards!

Muluken Chanie Agimas

ID: https://orcid.org/0000-0003-0808-124X

**Comments from review and editorial board**	**Author response**
**Editor's comment**	
put the sampling procedure in flow diagram<br><br>include limitations<br><br>look at your findings again and include additional recommendations	Dear Editor, It is a very interesting comment, and we accepted it, and the necessary amendment has been made accordingly. Regarding the limitation, it was written in the discussion. If it should be written separately, we did it.

**Reviewer one comments**	Author response
Abstract: The abstract reflect aspects of the study clearly: background, objectives, methods, results and conclusions, but in the methods part selecting method of model (what means “the lowest Akaike information criteria” the author should be make it clear explain). And author missed to leave educational status set as “significant predictors of emergency cesarean delivery” because, it wasn’t show association under AOR or multivariable analysis	Thank you, dear reviewer. You are right; we have made a mistake. We took the significant predictors for educational status in model I, but our best model was model III (mixed effect model). So the necessary correction has been made. Regarding the lowest Akaike information criteria, AIC estimates the relative amount of information lost by a given model: the less information a model loses, the higher the quality of that model. In estimating the amount of information lost by a model, AIC deals with the trade-off between the goodness of fit of the model and the simplicity of the model. Therefore, when we say the lowest Akaike’s information criteria (AIC), we mean that the best model was compared by AIC. The successive decrement of the AIC value in the models implies that the model best fits the data. Its AIC goodness of value was 695. A necessary amendment has been made to avoid this ambiguity.

**Introduction**: The study rationale satisfactorily described but as gaps: limited to describe , gaps and plan or purpose of study details and the author should address that it needs highlight revise	It is great, and you are right. Now the necessary amendment has been made accordingly.
**Methodology**: The study design appropriate and adequate for the plan of study, The sample size seems an appropriate but inadequate justified in case of set this value (11,022 included in analysis),the author should clarify the gaps and also needs highlight revise sampling technique. The author also missed to identify their number and professional of data collators, The author missed to state the exclusion criteria's that what action takes place in study during study period, Statistical analysis preferred analysis version is best and it is better to state the value of goodness of fit of the model.	Dear reviewer, It is a very interesting comment. We did not write everything reported from EDHS-2016 to make our manuscript more precise and to avoid detailed narrations in the manuscript. We want to provide a reference for more detail. That is why we describe only a few issues. Definitely, you are right; adding what you have asked makes the manuscript more informative. The necessary amendment has been made. Regarding the goodness of fit test value, it was displayed in the textual narration and in **Table 1.**
**Result**: The findings were presented logically with appropriate displays but, the author missed that the statement of bi-variable multilevel logistic regression at a p-value of <0.25 should be narrated under methods and also two lines sentences under the sub topic "Predictors of emergency cesarean delivery" should be stated under methods sub topic statistical analysis. In the title of tables and figure should be also incorporate study period of Ethiopian demographic health survey (EDHS) 2016data.	Thank you so much for your great comments. So we accept the comment and revise it.
**Discussion**: The key findings were stated, almost it seems well constructed and the findings also discussed in the light of previous evidence but it	I really appreciate your detailed view. After extensive searching,

shows the limitation of comparing findings with developing countries like African especially Sub-Saharan countries.	the prevalence of emergency caesarean delivery in the current study has been compared with previous African studies and those in other developing countries. But my significant variables like age, history of fistula, and rural residence were not significant in the previous African studies. Because of this, I have faced the challenge of comparing the risk factors with the previous African studies and comparable populations. As far as my searching goes, I cannot find a comparable study done in Ethiopia. But the risk factors for emergency caesarean delivery in this study were not adequately compared with the African studies. Because, as I mentioned earlier, my significant variables were not significant in the African studies.
Conclusion: The results justify the conclusion is fair.<br><br>Bibliography/References: The references appropriate and relevant, except a few references such as ref. NO9, 10 and 12 are later that needs replace with recent.<br><br>Others: Overall the paper presented logically and as a result, an author is advised to make some revisions then the paper will be become the best.	Thank you, dear reviewer, for your genuine comment. Now we update those references.
**Reviewer two comments**	**Authors response**

The manuscript, while addressing a good topic, appears to lack technical soundness. For instance, a statement in the manuscript mentions, "Specifically, in Ethiopia, the rate of all forms of cesarean delivery is from 8-37% (5, 13). But nothing is known about emergency cesarean delivery," which may not be accurate. This claim contradicts information found in a relevant paper accessible through this link: https://bmcpregnancychildbirth.biomedcentral.com/articles/10.1186/s12884-21-04266-7. Additionally, a comparison with another paper (file:///C:/Users/belac/Downloads/journal.pone.0282951%20) reveals significant differences in the author's contributions to the study.	Dear reviewer Thank you for your suggestion. Dear reviewer, in this study I try to address specifically **"emergency caesarean delivery"** and not the combined (all forms) of CS. Because low-income countries, including Ethiopia, are associated with low access to ANC services, and because of the three delays in accessing the delivery service and other problems, emergency caesarean delivery is expected. Previous studies conducted so far, including the article you suggested, "**Prevalence and associated factors of caesarian section in Ethiopia: a multilevel analysis of the 2019 Ethiopia Mini Demographic Health Survey,"** have considered women who have elected themselves for a planned caesarean delivery with non-medical indications. The article you suggested is not the only study conducted on CS in Ethiopia. There have been a lot of studies conducted on CS. But all the published articles were studied on the magnitude and risk factors for combined (all forms) of CS. But the magnitude and the risk factors of

	elective CS, CS without medical indication, and emergency CS are different. Combining these into one term, "CS," is broad and vague. It will not answer the question, What is a risk factor for a specific type of emergency CS? To apply a specific intervention, studies on specific types of CS have more importance. Generally, the article you suggested and other studies done in Ethiopia were conducted on the prevalence and risk factors of the combined (all forms) of caesarean delivery (CS), including CS without medical indication, elective CS, and CS for any reason. But a risk factor for elective CS may be different from a risk factor for CS without medical indication, and a risk factor for emergency CS is reasonably different from a risk factor for elective (without medical indications of CS). Furthermore, the risk factors for emergency CS are different from the risk factors for the combined (all-form) CS. So trying to identify a risk factor for all forms of CS may be misled to identify a risk factor for emergency CS. Previously published studies

	also failed to report the prevalence of emergency CS in particular. Specifically, identifying a risk factor for emergency CS is scientifically important for early assessment and screening practices to save the lives of women and newborns. Thus
The author should mention in details restrictions on publicly sharing data—e.g. participant privacy or use of data from a third party—those must be specified.	Dear reviewer, a necessary amendment has been made in the ethical consideration section.
Strongly advise the author to edit the grammar.	Dear reviewer honestly speaking, your comments provide an insight to revise my manuscript extensively to make it a more scientifically important paper. So I attentively see your comments and the necessary revisions you have made.
**Reviewer three comments**	**Author response**
**Abstract**<br>**Methods**	
1. Was the entire study sample of the EDHS 2016 considered for the study?	Dear reviewer, I used only women who gave birth prior to the survey, not all of the sample.
2. If not how, this sample (i.e., 110222) was selected?	It was not 110222, rather a total of 11,022 sample was included. The first enumeration areas were selected and then stratified into

	urban (202 enumeration areas) and rural (443 enumeration areas). Then, in each EA, a number of households were identified and selected. Then, from urban total households, 2355 women who gave birth prior to the survey were selected, compared to 8667 women from rural residence. For more detail, it was displayed in **Figure 1**.
3. What makes this study Multilevel?	The reason we used such a model was because of the hierarchical nature of the EDHS data and the possibility of considering a natural nesting of data.
4. "...The best model was selected based on the lowest Akaike information criteria..." So, what is the best model selected explain it?	Models were compared by AIC. The successive decrement of the AIC value in the models implies that the best fits the data. Based on the lowest AIC criteria, model III (the mixed effect model) was the best model. Its AIC goodness of value was 695 and its PCV was 79.2%.
**B. Results**<br><br>5. Are these micro-level or Macro-level variables?	It was micro level variables.
6. What about the Intraclass correlation (ICC) report? Have checked whether or not there are clustering of the cesarean section?	Dear reviewer, Though it was reported in **Table 1,** I forgot to mention in textual narration. An amendment has been made

	accordingly in the result section again.
**Introduction**<br>7. Line 51-52, "... According to the world health organization (WHO) recommendation, the cesarean delivery should be from 5-15%..." is this information about elective or emergency cesarean section?<br>8. What is the expected magnitude of Emergency cesarean delivery, according to WHO?<br>9. Line 65-67, if numerous studies were conducted, why you failed to mention a magnitude of cesarean section in Ethiopia?	The WHO CS recommendation is about all forms of CS. As far as my searching goes, I cannot find the WHO recommended value for an emergency Caesarean section. Dear reviewer, the magnitude of all (combined) forms of caesarean section is well addressed, so I can add the most recent figures of CS in Ethiopia. Because it is too hard to report all the findings in my article. Therefore, the manuscript has been revised with only the most recent data.
**Methods**<br>10. Line 75-77, better to update the regions of Ethiopia based on the recent classification?<br>11. Line 78, "... world speedometer..." Is it appropriate term? Worldometer? Is the report of worldometer valid and acceptable?<br>12. Line 81-83, were elective women for cesarean delivery, who undergone cesarean section two or more times eligible for this study?<br>13. Line 98, the sampling procedure is not clear? Is to mean multistage random sampling or what?	Dear reviewer, regarding the updated regions of Ethiopia, I have already stated. The newly emerged regions of Ethiopia are south-west Ethiopia, central Ethiopia, and the Sidama region. Therefore, I try to write by adding to the other nine previous regions. There is no additional region beyond what I have stated.<br><br>Regarding the worldometric report of the Ethiopian population, there has been no recent national census in Ethiopia. It has been more than

	10 years since Ethiopia conducted a national census. Therefore, there is no available estimate of the Ethiopian population. That is why I prefer to use the worldometer estimate.<br><br>Concerning the eligibility of the participants, we used the secondary data from EDHS 2016. So the EDHS data reported the emergency caesarean section delivery as “yes” and no." It was not clearly present or reported about women who underwent caesarean sections twice or more imes. Just they classify as “emergency caesarean delivery” when the women have a history of emergency caesarean delivery preceding the survey; otherwise, no for emergency caesarean delivery.<br><br>The EDHS used the multistage sampling technique, and so amendments have been made to the manuscript.
14. Line 99, it is not clear how a sample size of the study was determined?<br><br>15. Line 120, It is better if the best model selected was reported instead.<br><br>16. Line 126, before using the multilevel model, It is must to elaborate the clustering variable either in the operational definition or elsewhere?<br><br>17. Line 128, it is not clear which variables are considered as "X" or "Z".	Dear reviewer Everything you have mentioned is very interesting. Definitely, the method section lacks clarity. Therefore, revisions and corrections have been made to

	the sample size determination (using the schematic diagram in Figure 1), model selection, and clustering variables.<br><br>The XY variable represents that X is a level I (individual level) predictor and Y is a level II (community level) predictor.
**Result**<br><br>18. Line 147, before rushing to identification of predictors using multilevel binary logistic regression, testing of the appropriateness of the model required. So, the author must clarify the values of ICC, PCV, and AIC, that reported in the table 1. Additionally, it is not clear what model I, Model II, and Model III are? On the other way, the clustering variable is not clear to apply the model?<br><br>19. Line 153, what is the term "... After all..." means? Is it to mean that after doing Crude analysis (bivariate binary logistic regression)? It needs more detail explanations?	Thank you so much for your scientific contribution to making my paper suitable for publication. You are right; the result section missed reporting important findings. So corrections have been made in this section.<br><br>Dear reviewer, the word after all in line 153 means after conducting multivariable multilevel binary logistic regression. So to avoid this ambiguity, an amendment has been made to the section.
**Discussion**<br><br>20. Line 169-172, in spite of what you justified, do you think it is fair to compare Japan with Ethiopia?<br><br>21. Add more comparison and discuss it as this result is not well discussed?<br><br>22. In general your discussion is comparing incomparable population and results?<br><br>23. Line 175-176, how do you see comparing all type of cesarean section to emergency cesarean section (your study)?<br><br>24. what is the strengthen of this study?	Dear reviewer you are right comparison population should be comparable. The reason what I compare with Japan was to show how the emergency cesarean delivery is vary as compared to developed countries. But I feel that it is known fact that the emergency cesarean delivery service is vary in

	developed and developing countries. **Therefore based on your comment**, After extensive searching, the prevalence of emergency caesarean delivery in the current study has been compared with previous African studies and those in other developing countries. But my significant variables like age, history of fistula, and rural residence were not significant in the previous African studies. Because of this, I have faced the challenge of comparing the risk factors with the previous African studies and comparable populations. As far as my searching goes, I cannot find a comparable study done in Ethiopia. But the risk factors for emergency caesarean delivery in this study were not adequately compared with the African studies. Because, as I mentioned earlier, my significant variables were not significant in the African studies. Regarding comparing with all forms of Cesarean delivery, I feel it

	is illogical wrong and therefore necessary amendment has made. Whereas the strength of this study was using nationally (country-wide) representative sample provides a powerful evidence.
**Reviewer four comments**	**Author response**
Topic is not " SMART" and lacks novelty =revise it<br><br>>Data source is secondary and not current which might affects reliability and accuracy of the result<br><br>>Try to clarify your research methods and materials and significance of your study	Dear reviewer Regarding the novelty, as far as I can tell from my searching, there has been no research conducted specifically on emergency caesarean delivery. The previous studies in Ethiopia were conducted on the prevalence and risk factors of combined (all forms) caesarean delivery (CS), including CS without medical indication, elective CS, and CS for any reason. But a risk factor for elective CS may be different from a risk factor for CS without medical indication, and a risk factor for emergency CS is reasonably different from a risk factor for elective (without medical indications of CS). Furthermore, the risk factors for emergency CS are different from the risk factors for the combined (all-form) CS. So trying to identify a risk factor for all forms of CS may be misled to

	identify a risk factor for emergency CS. Previously published studies also failed to report the prevalence of emergency CS in particular. Specifically, identifying a risk factor for emergency CS is scientifically important for early assessment and screening practices to save the lives of women and newborns.<br><br>Thus,<br><br>Conducting studies on emergency caesarean delivery is a very important tool for screening practices and developing working guidelines for health professionals. Because of a lack of adequate evidence, there is no established protocol for health professionals to identify pregnant women early for emergency caesarean delivery and prompt management in Ethiopia.<br><br>Dear reviewer, this is a novel study. Really, I would like to assure you once again that this is an unaddressed research area. Based on my effort to conduct this study and my extensive revision of the

	manuscript, I hope you will be positive about my work.<br><br>Regarding the data source,<br><br>1. To assure the reliability and accuracy of EDHS 2016 data, it was collected through a validated questionnaire.<br>2. Frequent supervision was conducted during the data collection.<br>3. Careful and double-checking of data during data entry.<br>4. To be more representative, a sampling weight and proportional allocation technique were used.<br>5. Pretests and training were conducted to assure the reliability and accuracy of the data.<br>6.<br>7. It consisted of in-class training, biomarker training, and field practice days. The field practice was conducted in clusters surrounding Bishoftu that were not included in the 2016 EDHS sample.<br>8.<br>9. A total of 60 trainees attended the pretest. The trainees had experience with household surveys.<br>10.<br>11. Having been involved in either previous Ethiopia DHS surveys or other similar surveys. Following the field

	practice, a debriefing session was held with the pretest field staff, and modifications to the questionnaires were made based on lessons drawn from the exercise.<br><br>So I used the data collected in the above way.<br><br>Otherwise, like other secondary data source studies, my study also shares the limitations of secondary data sources. I consider this a limitation of the study.<br><br>**Regarding "the study is not current";**<br><br>This study was aimed at serving as a baseline for future studies. There is no study elsewhere in Ethiopia specifically on emergency caesarean delivery risk factors and their magnitude. Even in the EDHS 2019 data set, the emergency caesarean delivery was not reported because EDHS-2019 is a ini-EDHS. That is why I used EDHS 20116 data for this study because it is the only recent national data source available for emergency caesarean delivery. Additionally, till now, EDHS 2021 data has not been reported for further research.<br><br>For your comment **"Try to clarify your research methods and materials and significance of your study"**<br><br>Even though the comment is general, you are right; I feel that the method section lacks clarity. Therefore, based on your comment, other reviewers comments, and my

	observation, extensive revision has been made in this section. The change is highlighted or colored. The significance of the study is also included in the introduction section.<br><br>Regarding the SMART of the title<br><br>My title was **specific**= emergency cesarean delivery among women who give birth<br><br>**Measurable**= emergency cesarean delivery is easily measurable<br><br>**Achievable**= my research is achievable with cost effective use of secondary data source.<br><br>**Time bounded:** My title is time bounded which use the data collected in 2016 and it is **realistic**.<br><br>May be my title lacks the source population, which was not mentioned, and revisions have been made as follows.<br><br>**"Prevalence of emergency cesarean delivery and its predictors among women who give birth in Ethiopia using further analysis of EDHS 2016 data: A mixed effect model"**

Thank you, dear editor and reviewers, for your great scientific contribution!
