# Supplementary material for: Prevalence of emergency caesarean delivery and its predictors among women who give birth in Ethiopia using further analysis of EDHS 2016 data: A mixed effect model
Source: PLoS One. 2024 May 16;19(5):e0300528. doi: 10.1371/journal.pone.0300528 (PMC11098499; doi:10.1371/journal.pone.0300528)
Supplement: Attachment — Submitted filename: PloseOne.docx [file pone.0300528.s001.docx]

**Abstract**

**Methods**

1. Was the entire study sample of the EDHS 2016 considered for the study?
2. If not how, this sample (i.e., 110222) was selected?
3. What makes this study Multilevel?
4. “...The best model was selected based on the lowest Akaike information criteria..." So, what is the best model selected explain it?

**Results**

5. Are these micro-level or Macro-level variables?
6. What about the intraclass correlation (ICC) report? Have checked whether or not there are clustering of the cesarean section?

**Main Body**

**Introduction**

7. Line 51-52, "... According to the world health organization (WHO) recommendation, the cesarean delivery should be from 5-15%..." is this information about elective or emergency cesarean section?
8. What is the expected magnitude of Emergency cesarean delivery, according to WHO?
9. Line 65-67, if numerous studies were conducted, why you failed to mention a magnitude of cesarean section in Ethiopia?

**Methods**

10. Line 75-77, better to update the regions of Ethiopia based on the recent classification?
11. Line 78, "... world speedometer..." Is it appropriate term? Worldometer? Is the report of worldometer valid and acceptable?
12. Line 81-83, were elective women for cesarean delivery, who undergone cesarean section two or more times eligible for this study?
13. Line 98, the sampling procedure is not clear? Is to mean multistage random sampling or what?
14. Line 99, it is not clear how a sample size of the study was determined?
15. Line 120, It is better if the best model selected was reported instead.
16. Line 126, before using the multilevel model, It is must to elaborate the clustering variable either in the operational definition or elsewhere?
17. Line 128, it is not clear which variables are considered as "X" or "Z".

**Result**

18. Line 147, before rushing to identification of predictors using multilevel binary logistic regression, testing of the appropriateness of the model required. So, the author must clarify the values of ICC, PCV, and AIC, that reported in the table 1. Additionally, it is not clear what model I, Model II, and Model III are? On the other way, the clustering variable is not clear to apply the model?
19. Line 153, what is the term "... After all..." means? Is it to mean that after doing Crude analysis (bivariate binary logistic regression)? It needs more detail explanations?

**Discussion**

20. Line 169-172, in spite of what you justified, do you think it is fair to compare Japan with Ethiopia?
21. Add more comparison and discuss it as this result is not well discussed?
22. In general your discussion is comparing incomparable population and results?
23. Line 175-176, how do you see comparing all type of cesarean section to emergency cesarean section (your study)?
24. What is the strengthen of this study?
